# Test-retest repeatability of intravoxel incoherent motion (IVIM) measurements in the cervical cord

**DOI:** 10.1162/imag_a_00468

**Published:** 2025-02-10

**Authors:** Anna Lebret, Simon Lévy, Patrick Freund, Virginie Callot, Maryam Seif

**Affiliations:** Spinal Cord Injury Center, Balgrist University Hospital, Zurich, Switzerland; Aix-Marseille Univ, CNRS, CRMBM, Marseille, France; APHM, Hôpital Universitaire Timone, CEMEREM, Marseille, France; MR Research Collaborations, Siemens Healthcare Pty Ltd, Melbourne, Australia; Max Planck Institute for Human Cognitive and Brain Sciences, Leipzig, Germany

**Keywords:** IVIM, test-retest measurements, repeatability, cervical spinal cord MRI, perfusion mapping

## Abstract

Intravoxel incoherent motion (IVIM) measurements allow to probe tissue microcirculation non-invasively. Spinal cord perfusion has been shown to be altered following different neurological pathologies. A non-invasive imaging protocol to assess perfusion in the cervical cord is, therefore, clinically relevant. This work aimed at assessing the reliability of IVIM parameters sensitive to perfusion changes in the cervical cord by determining the test-retest variability across subjects and different post-processing fitting algorithms. IVIM test-retest scans were acquired in the cervical cord (C1-C3) of 10 healthy subjects on a 3T MRI scanner, with a 15-minute break in-between. IVIM parameters, including microvascular volume fraction (F), pseudo-diffusion coefficient ($D^{*}$), blood flow-related coefficient ($F\cdot D^{*}$), and diffusion coefficient (D), were derived using voxel-wise and region of interest (ROI)-wise fits. The reliability of each IVIM parameter was determined with coefficients of variation (CV), intraclass correlation coefficients (ICC), Bland-Altman analysis, and linear regression. To assess the effects of the different fitting approaches, a two-way repeated-measures analysis of variance (ANOVA) was conducted on the CVs calculated across fitting algorithms. Mean CVs of IVIM parameters calculated across subjects using the voxel-wise fit were lower in the white matter (WM) and grey matter (GM): (WM: 2.6% to 15.6%; GM: 2.2% to 16.4%) compared with those calculated using the ROI-wise fit approach (WM: 4.5% to 32.2%; GM: 3.4% to 53.4%). The voxel-wise fit in the WM yielded higher ICC values (good-to-excellent, 0.71–0.97) compared to the ROI-wise fit approach (poor-to-excellent, 0.49–0.90). IVIM parameters, derived using the voxel-wise fitting approach, demonstrated a high reliability in the cervical cord. Results highlight the high variability of IVIM parameter values depending on the fitting approach, underlining the importance of characterizing the reliability of IVIM acquisition and fitting configuration in the relevant organ of interest. Robust IVIM metrics using a voxel-wise one-step approach, observed across scans and subjects, can facilitate studies targeting perfusion impairment and pave the way to future clinical trials assessing perfusion impairment as a potential quantitative biomarker.

## 1 Introduction

Intravoxel incoherent motion (IVIM) is a non-invasive quantitative MRI technique used to indirectly assess tissue micro-perfusion by determining the contributions of microcirculation and thermal diffusion to the MR signal decay (Le Bihan et al., 1988). It is based on the circulation of blood water molecules in the capillary bed as a pseudo-Brownian diffusion process, under the assumption of randomly oriented capillaries in the tissue or rapidly changing blood in aligned capillaries (Le Bihan et al., 1988). IVIM MRI provides parameters indirectly sensitive to the microvascular volume (F), blood velocity ($D^{*}$), blood flow ($F\cdot D^{*}$), and tissue diffusion (D) (Le Bihan & Turner, 1992). Previous reports have shown that IVIM is sensitive to changes in tissue perfusion (Callot et al., 2003;Guiu et al., 2012;Lemke et al., 2009;Luciani et al., 2008;Spinner et al., 2021;Woo et al., 2014). This technique has thereby potential to study perfusion impairment and ensuing ischemia occurring in the damaged spinal cord in a variety of neurodegenerative diseases (Ellingson et al., 2019;Hernandez-Gerez et al., 2019). Characterization of perfusion dysfunction is crucial to better understand its role in the disease progression and severity. Previous studies have shown that different fitting approaches of the IVIM model (Lévy et al., 2020;Orton et al., 2014) can result in a large variability by applying different algorithms in organs of various perfusion levels (Barbieri et al., 2016;Meeus et al., 2017;Spinner et al., 2021;Wang et al., 2021). The two most common fitting methods are the “two-step” or “segmented” approach and the “one-step” approach. These methods differ in the order of parameters estimation and have been previously explored in IVIM studies across various organs (Barbieri et al., 2016;Cho et al., 2015;Lévy et al., 2020;Mozumder et al., 2018). On the other hand, IVIM parameters in a specific region of interest (ROI) can be derived either using a voxel-wise fit approach (followed by averaging across the ROI) or an ROI-wise fit (fitting the average MR signal across the ROI). Thus, it is of great relevance to determine which fitting approach in IVIM modeling yields the most reliable outcomes in terms of robustness to noise and variability. While IVIM MRI has already been applied in the cervical cord (Lévy et al., 2020), its reliability in this organ remains understudied at 3T for potential clinical applications. Importantly, cervical cord MRI measurements are limited by several challenges, including a low signal-to-noise ratio due to RF coil coverage compared to other organs such as the brain (Cohen-Adad, 2018;Stroman et al., 2014;Wheeler-Kingshott et al., 2014), motion artifacts from both physiological and spinal cord-related movements (Kharbanda et al., 2006), and susceptibility artifacts induced by B_0_inhomogeneities of different tissue types near the spinal cord causing distortions and signal drop (Cohen-Adad, 2018). Determining IVIM parameters’ reliability in the cervical cord is, therefore, essential to quantify the minimum detectable pathophysiological changes and substantiate IVIM findings in future studies. The objective of this study was, thus, to determine the reliability of IVIM parameters calculated by the one-step and two-step fitting approach in the cervical cord, based on both voxel-wise and ROI-wise fitting methods.

## 2 Methods

### 2.1 Standard protocol approvals, registrations, and participant consents

The study protocol was designed according to the Declaration of Helsinki and approved by the local Ethics Committee (EK-2018-00937). Informed written consent was obtained from each participant before study enrolment.

### 2.2 Participants and MR acquisition

Ten healthy controls (mean age ± SD: 30.0 ± 5.4 years, 4 females) were scanned twice, with a 15-minute break out of the scanner with repositioning and shim reset between the sessions. The exclusion criteria for the study enrolment were prior operation to the cervical spine, MRI contraindication, pre-existing neurological condition, and age under 18 or above 75 years old. MRI data were acquired on a 3T MRI scanner (MAGNETOM Prisma, Siemens Healthineers, Erlangen, Germany) with a Siemens Healthineers 64-channel head and neck radio-frequency coil. A stifneck collar (Laerdal Medicals, Norway) was used on every subject to minimize motion artifacts in the inferior-superior direction.

### 2.3 Image acquisition protocol

Study participants underwent a protocol composed of a sagittal T2-weighted turbo spin echo (TSE) sequence to localize the cervical levels, an axial T2*-weighted 3D multi-echo gradient-echo sequence (Büeler et al., 2022) to segment grey and white matter, and a vendor product cardiac-gated (Cohen-Adad et al., 2021) IVIM axial 2D-RF diffusion-weighted spin-echo EPI ZOOMit (Zonally-magnified Oblique Multislice) sequence with a trigger delay of 100 ms (using the Siemens standard pulse oximeter), a 34 × 108 matrix size, 9 slices and 3 concatenations (3 slices/cardiac cycle), and 14 b-values ranging from 0 to 650 s/mm^2^with an increment of 50 s/mm^2^([0; 50; 100; 150; 200; 250; 300; 350; 400; 450; 500; 550; 600; 650]) (Lévy et al., 2021) with 20 repetitions per b-value in three in-plane diffusion-encoding directions to assess perfusion (60deg, 180deg, -60deg). Diffusion-encoding directions were restricted to the transverse plane, as the estimation of IVIM parameters along the inferior-superior spinal axis (z-axis of the scanner) has been shown previously to be compromised by the high water diffusivity coefficient at high b-values in that direction and therefore not reliable (Lévy et al., 2021). The IVIM acquisitions were split into forward and reverse phase-encoding directions for subsequent distortion correction (10 repetitions/b-value in each phase-encoding direction). The actual acquisition time depended on the subject’s heartbeat and varied between 6 and 10 minutes for each phase-encoding direction. All scans were acquired in the cervical cord covering C1-C3 levels. The total nominal acquisition time per scan-rescan session was ca. 39 minutes (depending on individual heart rate). MRI parameters of the different sequences are reported inTable 1.

**Table 1.** MRI scan parameters of the protocol sequences.

Sequence parameters	Sagittal T2w (TSE)	Axial T2*w (meGRE)	Axial IVIM (SE-EPI)
TE [ms]	84.0	6.85, 10.85, 14.85, 18.85, 22.85	58.0
TR [ms]	3500	38.0	Nominal = 600, Concatenations = 3 Effective = 3 x subject’s cardiac cycle
Flip angle [°]	160	8	90
FOV [mm ^2^ ]	220 × 220	192 × 192	101 × 31.8
In-plane resolution [mm ^2^ ]	0.3 × 0.3	0.5 × 0.5	0.9 × 0.9
Slice thickness [mm]	2.5	5.0	5.0
Number of slices	20	16	9
Readout bandwidth	260 Hz/pixel	260 Hz/pixel	1402 Hz/pixel
Nominal acquisition time [min]	1:47	6:29	5:08 ( *per phase-encoding direction* )
Parallel imaging	GRAPPA	GRAPPA	None
Acceleration factor (iPAT) PE	2	2	-

GRAPPA: generalized autocalibration partially parallel acquisition.

### 2.4 Image processing

IVIM images were processed using custom-made scripts of the IVIM toolbox (Lévy et al., 2020), using functions from the Spinal Cord Toolbox (SCT) (De Leener et al., 2017). The processing steps consisted of denoising of each phase-encoding direction using a local principal component analysis algorithm (Manjón et al., 2013), removal of Gibbs artefacts using the local sub-voxel-shifts method (Kellner et al., 2016;Perrone et al., 2015) (*DIPY*(Garyfallidis et al., 2014)), and finally motion (*sct_dmri_moco*(De Leener et al., 2017)) and EPI-related distortion correction (*FSL topup*(Andersson et al., 2003)).

Prior to fitting the IVIM signal, voxel-wise SNR maps were determined to evaluate data quality and compare SNR values to previous investigations assessing required SNR for model accuracy (Lévy et al., 2020). The maps were generated based on the distortion-corrected images acquired at b_0_and calculated as the ratio of the mean signal across repetitions to the standard deviation across repetitions, in a voxel-wise manner (Lévy et al., 2020;Reeder et al., 2005). To represent the signal fed to the fitting algorithm, the SNR values were multiplied by a factor$\surd {\text{N}}_{\text{rep}}$(representing the number of repetitions). SNR values were extracted in subject space within the eroded spinal cord mask (to minimize partial volume effect with the CSF) and averaged across C1-C3 levels and subjects for each scanning session.

#### 2.4.1 IVIM signal fitting

The IVIM signal is commonly described by the following biexponential decay model (Le Bihan et al., 1988):

$$S\left(b\right)=S_{0}e^{-bD}\left[Fe^{-bD^{*}}\text{​}+1-F\right]$$

whereFis the microvascular volume fraction,$D^{*}$is the pseudo-diffusion coefficient (proportional to blood velocity in the capillaries(Le Bihan & Turner, 1992)), andDis the diffusion coefficient. Furthermore,$F\cdot D^{*}$can be proportionally related to blood flow (Le Bihan & Turner, 1992).

To assess the robustness of the fit and the reliability of IVIM measurements obtained with different fitting approaches, IVIM maps were calculated with a voxel-wise and ROI-wise fit, using two different standard algorithms, namely a constrained one-step method and a constrained two-step “segmented” method (Lévy et al., 2020).

Furthermore, maps of R^2^were computed during the fit for each fitting configuration to provide indication about the quality of the fitting procedure.

#### 2.4.2 One-step and two-step algorithms

Briefly*,*in the one-step algorithm (Pekar et al., 1992), a prior estimation ofDwas performed for boundaries determination using only a mono-exponential fit based on b-values above 400 [s/mm^2^] and using the*Conjugate Gradient*optimization method. The fit of all parameters was then performed based on all b-values (0 to 650 [s/mm^2^]) using the*Differential Evolution*optimization method (Storn & Price, 1997) (to escape local optima) and constrainingDbetween 1.5 × 10^-4^[mm^2^/s] and 54 × 10^-4^[mm^2^/s]. The other parameters were confined in a range of values published in the literature for the brain:F[%]: [0; 20];$D^{*}$[mm^2^/s ×10^-3^]: [0.3; 50]. In a final step, fine-tuning of the parameters with tighter boundaries (constraining the parameter estimation to 95% - 105% of the previously estimated values) was conducted.

In the two-step method, the IVIM parameters are estimated in two different steps.Dwas first estimated using a mono-exponential fit based on b-values above 400 [s/mm^2^]. The fit ofFand$D^{*}$was then performed on all b-values as described for the one-step method, keepingDfixed to the value estimated in the first step. The last step consisted of fine-tuning ofFand$D^{*}$, constraining the value estimation with tighter boundaries (95%–105% of the estimation obtained at the previous step), whileDremained fixed to its initial estimation.

#### 2.4.3 Voxel-wise and ROI-wise fits

For the voxel-wise approach, the IVIM signal was fitted (using the one-step and two-step algorithms) in each voxel of the spinal cord segmentation (produced with*sct_deepseg_sc*(Gros et al., 2019)), resulting in voxel-wise IVIM maps in each diffusion-encoding direction.

White matter and grey matter were investigated separately in the ROI-wise approach. Probabilistic masks were calculated from the white matter atlas registered to the diffusion images (generated during the registration step, see next section) and eroded at the periphery of the spinal cord to avoid partial volume effect with the cerebrospinal fluid (CSF). The signal value in each ROI was obtained by calculating the weighted average of the diffusion signal values in each voxel with the probability value of the probabilistic mask in that voxel, per slice and per b-value. This ROI-wise signal decay was then fitted (using either the one-step or two-step algorithms), resulting in ROI-wise IVIM values per slice in each diffusion-encoding direction.

#### 2.4.4 Registration to template

**Fig. 1.**
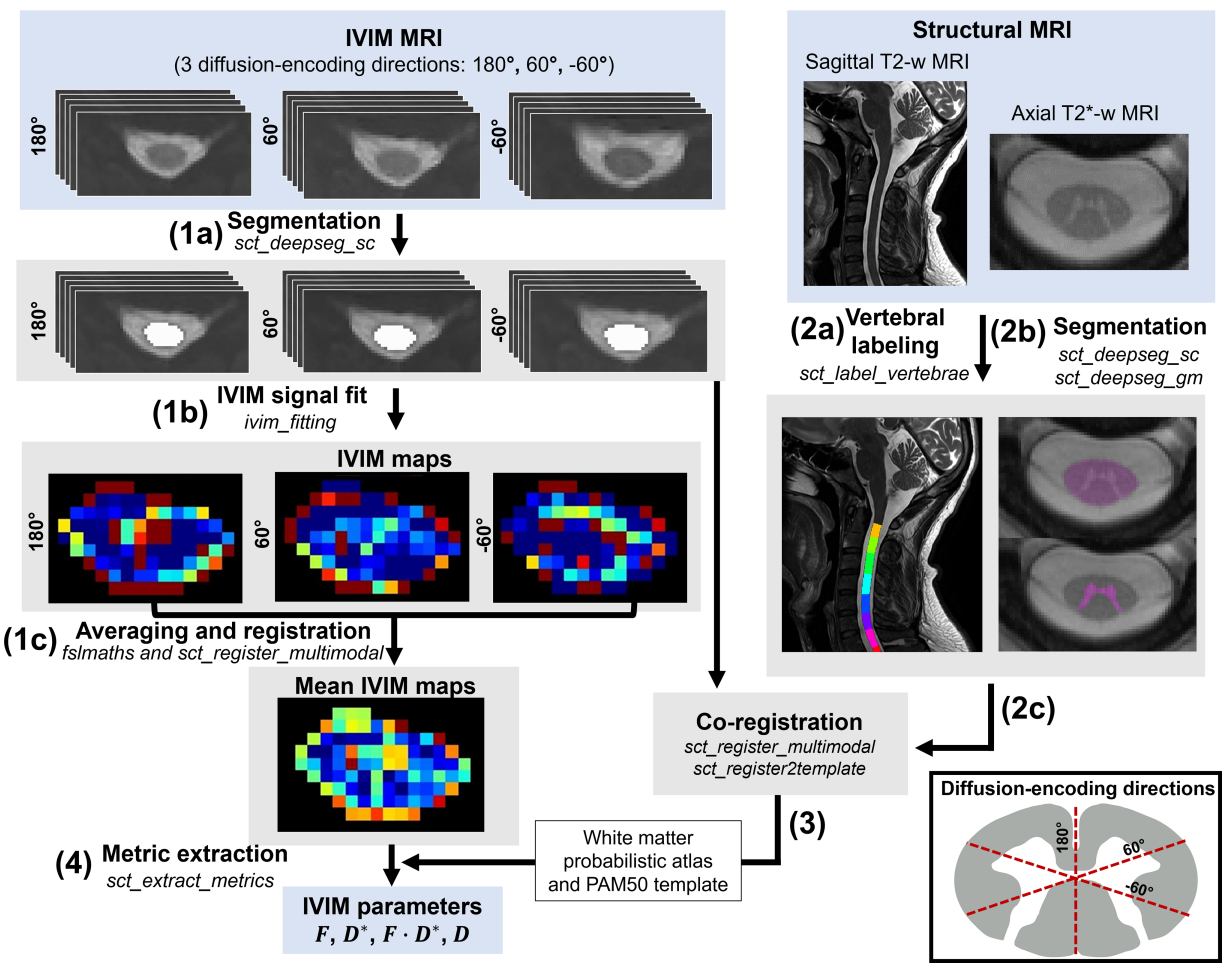
IVIM post-processing pipeline using the IVIM toolbox (Lévy et al., 2020). IVIM MRI: (1a) segmentation of the spinal cord is conducted on each individual diffusion-encoding direction (i.e., 180°, 60°, -60°). (1b) IVIM maps (hereFmaps are shown for illustration) are generated from the distortion-corrected diffusion images using the voxels provided by the cord segmentations in each of the three diffusion-encoding directions and then (1c) averaged across directions (after registration of the IVIM images to the 180° diffusion-encoding direction). Structural MRI: (2a) vertebral labeling on sagittal T2-weighted MRI and (2b) segmented spinal cord and grey matter on the axial T2*-weighted images are obtained and (2c) further used for the co-registering to the IVIM images. As a final step, (4) IVIM parameters are extracted in each slice in the white and grey matter after (3) registration of the maps to the PAM50 template and its white matter atlas, using warping fields derived from the co-registration of the IVIM images to the structural images (at step 2c). Note that the averaging across repetitions per b-value and the distortion correction are not illustrated here and occurred before the registration pipeline. The averaging across slices corresponding to C1-C3 levels (from which the test-retest differences, CV and ICC were then derived) is not illustrated either. Visualization of the diffusion-encoding directions is provided in the box in the bottom-right corner.

The white and grey matter probabilistic masks were needed to generate an average signal value per slice in the ROI-wise approach and extract the IVIM metrics. Thus, the IVIM images and maps were registered to the PAM50 template (De Leener et al., 2018) and white matter atlas (Lévy et al., 2015) with an intermediate registration step performed on T2*-weighted images to account for the subject’s variability in the grey matter shape (Fig. 1, step 3). The IVIM maps in the 60° and -60° diffusion-encoding directions were registered to the 180° one and then averaged across all directions (Fig. 1, step 1C). This approach was favored over the alternative method, which involves registering the IVIM images of different directions to each other prior to fitting the average IVIM signal values (across directions). The rationale behind this choice was related to the dependency of the water diffusivity on the tissue microstructure, leading to variations in theDcoefficient based on the diffusion-encoding direction. Since the estimation of the IVIM parameters depends on the value ofDduring the fitting process, fitting the IVIM model on an average signal value across directions can blend the direction-dependent effects ofDon the IVIM parameters. This can then lead to deviations of the signal decay from the representation provided by the model. Therefore, the IVIM signal fit was performed on the individual directions. Finally, the IVIM parameters were extracted from the individual maps (averaged across directions), in subject space, averaged across C1-C3 vertebral segments, in the ROIs, for both the ROI-wise and voxel-wise approaches (Fig. 1, step 4). The regions of interest were eroded before metric extraction at the cord periphery, with*fslmaths*from the FSL library (v6.0.5, Analysis Group, FMRIB, Oxford, UK) (Jenkinson et al., 2012), using a sphere kernel of radius 1, to minimize partial volume effect with the CSF. An overview of the processing pipeline is shown inFigure 1. The R^2^values were extracted from the maps in each diffusion-encoding direction in subject space and averaged across C1-C3 levels and subjects.

### 2.5 Test-retest statistical analysis

The test-retest repeatability was determined for the four fitting approaches investigated (voxel-wise one-step, voxel-wise two-step, ROI-wise one-step, and ROI-wise two-step), using the following standard reliability measures:

The within-subject (ws) coefficients of variation (CV), estimating the variability across two scans for each subject, were calculated by the standard deviation ($\sigma_{ws}$) over the mean ($\mu_{ws}$) across both scanning sessions for each subject:

$$CV\left(ROI,subj,parameter\right)=\frac{\sigma_{ws}}{\mu_{ws}}*100$$

where*ROI*represents the different regions of interest,*subj*is the subject participating in the study, and*parameter*is each parameter of the IVIM model (F,$D^{*}$,$F\cdot D^{*}$, and*D*). To assess the effect of different fitting approaches (voxel-wise vs. ROI-wise) and fitting algorithms (one-step vs. two-step) on the CVs, a two-way ANOVA was performed. The dependent variable was the CVs values, while the independent variables were the different fitting approaches and the fitting algorithms as the two categorical variables. The analysis was within each ROI with a significance level set at α = 0.05.

The intraclass correlation coefficient (ICC) was applied to determine the test-retest reliability as a ratio of true variance (between the subjects) over the true variance plus the variance due to measurement errors (Koo & Li, 2016). The ICC implementation was based on MATLAB (version 9.13.0 (R2022b), the MathWorks, Inc., case 3A, absolute agreement) (Salarian, 2023) and the definition of McGraw and Wong (McGraw & Wong, 1996) for each parameter in each ROI. For the interpretation of the ICC values, we used the common scale introduced byCicchetti (1994),Cicchetti & Sparrow (1981), andFleiss (1981):*poor<0.4<fair<0.6<good<0.75<excellent*.

Next, a Bland-Altman analysis was conducted to define the mean difference between the sessions (i.e., bias) against the average value across sessions, along with the 95% limits of agreements (average difference ± 1.96 standard deviation of the mean difference).

Finally, Pearson linear correlations were performed between values obtained in the first and second sessions. A better test-retest repeatability is, therefore, represented by lower CVs, higher ICC, lower bias, and narrower limits of agreements in Bland-Altman analysis. Statistical analysis was performed using R software (version 4.1.2, R Core Team (R Core Team, n.d.)), aside from the ICC computation done in MATLAB.

## 3 Results

### 3.1 SNR values and quality of the fit in individual diffusion-encoding directions

SNR values remained similar across the various diffusion-encoding directions and scanning sessions.Table 2presents the mean, standard deviation, minimum, and maximum SNR values averaged across C1-C3 levels and subjects on the lowest b-value images on the distortion-corrected images (input of the fitting algorithm). The values are reported for each of the three diffusion-encoding directions and each scanning session.

**Table 2.** Mean and standard deviation of SNR values at b_0_of the distortion-corrected IVIM images within the eroded spinal cord mask, averaged across C1-C3 levels and subjects, and value ranges, for each diffusion-encoding direction (180°, 60°, and -60°) and scanning session separately.

Session 1			
	180°	60°	-60°
Mean ± SD	123.49 ± 37.20	118.65 ± 24.24	116.36 ± 29.18
(Min–Max)	52.46–177.44	84.65–159.05	69.16–165.11

Session 2			
	180°	60°	-60°
Mean ± SD	116.49 ± 38.18	142.81 ± 30.52	110.36 ± 28.32
(Min–Max)	61.91–200.14	97.88–186.51	83.90–165.49

The ROI-wise fit yielded better R^2^for each diffusion-encoding direction compared to the voxel-wise fit, using either the one-step or two-step approach. The two-step approach led to worse R^2^consistently, for both voxel-wise and ROI-wise fits. The mean and SD values of R^2^(averaged across subjects) are reported inSupplementary Table 1.

### 3.2 Mean IVIM values in the cervical cord

**Fig. 2.**
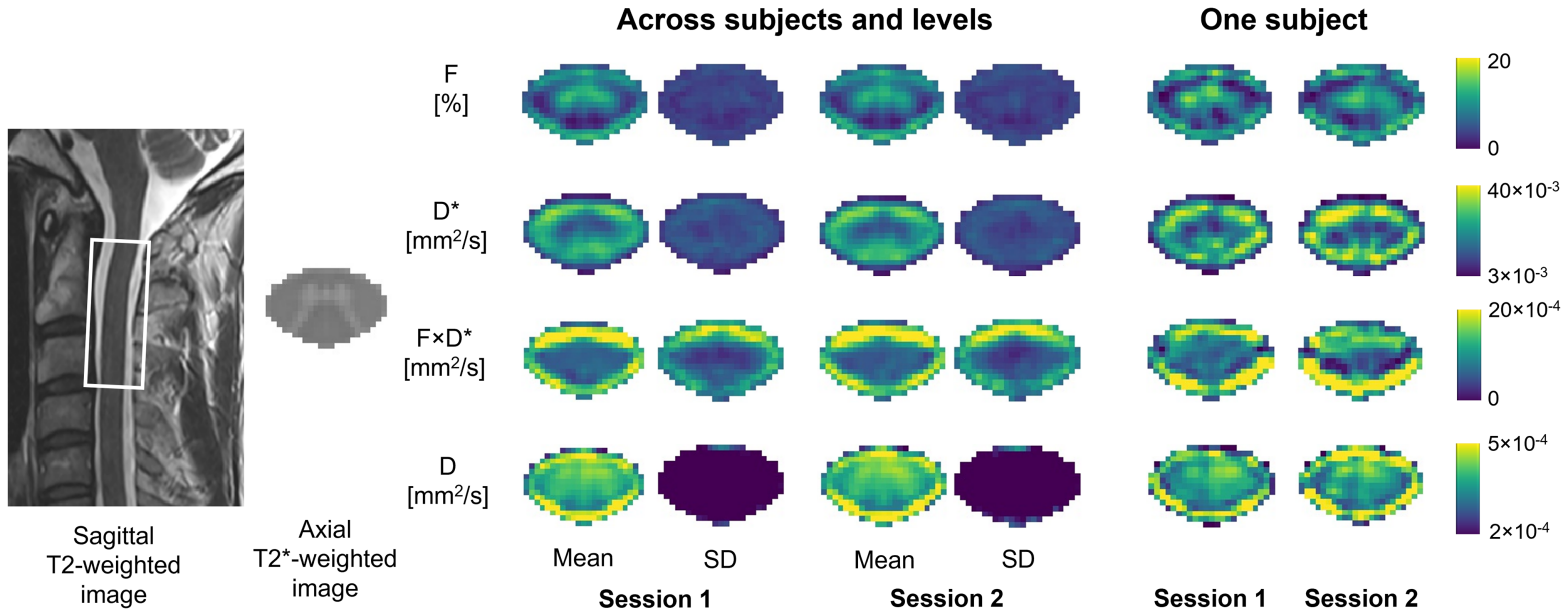
Voxel-wise one-step fit mean and standard deviation (SD) maps of the IVIM parameters (microvascular fraction (F), blood velocity-related coefficient ($D^{*}$), blood flow-related coefficient ($F\cdot D^{*}$), and diffusion coefficient (D)) in the template space, shown across subjects and for one subject, for both scanning sessions. Maps are averaged across C1-C3 vertebral levels and diffusion-encoding directions.

Figure 2shows IVIM maps (F,$D^{*}$,$F\cdot D^{*}$, andD) calculated in the voxel-wise fit using the one-step algorithm, averaged across subjects and C1-C3 levels. The microvascular volume fractionFappeared distinctly higher in the anterior and intermediate parts of the grey matter and highlights the greater vascularization of this structure of the spinal cord. The mean and standard deviation maps across subjects showed high similarity between the two sessions compared to the single subject maps, indicating a qualitatively good repeatability of the IVIM parameters averaged at the group level. The mean values of each IVIM parameter, averaged across C1-C3 levels, in the white and grey matter calculated with the voxel-wise and ROI-wise fits in each session are reported inTable 3.

**Table 3.** Mean and standard deviation values of IVIM parameters across participants.

(A) Voxel-wise												
	F [%]			$D^{*}$ [mm ^2^ /s ×10 ^-3^ ]			$F\cdot D^{*}$ [mm ^2^ /s ×10 ^-4^ ]			D [mm ^2^ /s ×10 ^-4^ ]		
*One-step*	S1	S2	Δ [%]	S1	S2	Δ [%]	S1	S2	Δ [%]	S1	S2	Δ [%]
WM	5.45 ± 0.75	5.49 ± 0.94	11.18	24.41 ± 2.61	24.32 ± 2.89	4.39	10.16 ± 2.67	10.32 ± 3.52	11.66	3.74 ± 0.49	3.77 ± 0.55	3.73
GM	9.61 ± 1.55	9.71 ± 1.39	16.98	17.14 ± 2.89	16.82 ± 3.45	12.77	8.48 ± 2.08	8.63 ± 2.17	12.12	4.17 ± 0.42	4.17 ± 0.48	3.08

	F [%]			$D^{*}$ [mm ^2^ /s ×10 ^-3^ ]			$F\cdot D^{*}$ [mm ^2^ /s ×10 ^-4^ ]			D [mm ^2^ /s ×10 ^-4^ ]		
*Two-step*	S1	S2	Δ [%]	S1	S2	Δ [%]	S1	S2	Δ [%]	S1	S2	Δ [%]
WM	4.03 ± 1.05	4.18 ± 1.29	23.67	24.38 ± 3.64	24.07 ± 3.20	8.57	7.50 ± 2.28	7.62 ± 2.85	11.28	4.23 ± 0.46	4.25 ± 0.49	5.74
GM	6.60 ± 1.40	6.80 ± 1.53	21.93	15.47 ± 3.94	15.54 ± 3.54	24.24	6.74 ± 1.88	6.78 ± 1.76	13.94	4.62 ± 0.40	4.62 ± 0.45	4.68

(B) ROI-wise												
	F [%]			$D^{*}$ [mm ^2^ /s ×10 ^-3^ ]			$F\cdot D^{*}$ [mm ^2^ /s ×10 ^-4^ ]			D [mm ^2^ /s ×10 ^-4^ ]		
*One-step*	S1	S2	Δ [%]	S1	S2	Δ [%]	S1	S2	Δ [%]	S1	S2	Δ [%]
WM	2.66 ± 1.09	2.76 ± 1.95	46.99	29.21 ± 10.39	29.57 ± 8.18	26.77	4.29 ± 2.49	5.02 ± 2.56	37.77	3.82 ± 0.48	3.88 ± 0.57	6.50
GM	11.45 ± 4.82	11.03 ± 3.25	40.05	11.68 ± 9.19	13.56 ± 8.39	100.48	4.62 ± 2.11	4.64 ± 1.54	26.27	3.98 ± 0.41	4.04 ± 0.34	8.99

	F [%]			$D^{*}$ [mm ^2^ /s ×10 ^-3^ ]			$F\cdot D^{*}$ [mm ^2^ /s ×10 ^-4^ ]			D [mm ^2^ /s ×10 ^-4^ ]		
*Two-step*	S1	S2	Δ [%]	S1	S2	Δ [%]	S1	S2	Δ [%]	S1	S2	Δ [%]
WM	1.73 ± 0.91	2.16 ± 1.27	57.07	24.20 ± 12.48	21.23 ± 8.96	38.16	2.63 ± 1.53	2.91 ± 2.28	43.24	4.00 ± 0.46	4.05 ± 0.48	7.10
GM	5.08 ± 1.21	5.29 ± 1.66	27.80	9.07 ± 7.41	10.55 ± 7.91	120.76	3.48 ± 2.28	3.10 ± 1.03	44.53	4.60 ± 0.42	4.59 ± 0.42	4.83

Values of IVIM parameters obtained in the white matter (WM) and grey matter (GM) for both sessions and mean (averaged across subjects) of the relative differences between the average values of the two sessions, using the one-step and two-step algorithms with (A) the voxel-wise fit and (B) the ROI-wise fit. S1: Session 1; S2: Session 2; Δ: average (across subjects) of the relative differences (session 1–session 2) in percentage of session 1.

### 3.3 Test-retest repeatability of IVIM parameters

#### 3.3.1 Comparing voxel-wise with ROI-wise fitting approaches

The within-subject CVs and the ICC calculated applying the voxel-wise and ROI-wise fits are reported inTable 4. The two-way ANOVA analysis resulted in a statistically significant difference across the mean of CVs across the different fitting approaches (voxel-wise vs. ROI-wise), where CVs calculated from the voxel-wise fitting approach were significantly lower compared to the ROI-wise fit (WM:F: p = 0.001,$D^{*}$: p = 5.3e-5,$F\cdot D^{*}$: p = 0.005; GM:$D^{*}$: 0.0003,$F\cdot D^{*}$: p = 0.002,D: p = 0.01), except forDin the white matter (p = 0.1) and forFin the grey matter (p = 0.07).

**Table 4.** Test-retest repeatability of IVIM parameters.

(A) Coefficients of variation (CV): mean and standard deviation values (across participants) calculated with the voxel-wise and ROI-wise fits, using the one-step and two-step algorithms in the white and grey matter.								
White matter								
	F		$D^{*}$		$F\cdot D^{*}$		D	
CV [%]	One-step	Two-step	One-step	Two-step	One-step	Two-step	One-step	Two-step
Voxel-wise	7.56 ± 6.59	15.57 ± 11.53	3.09 ± 2.13	6.08 ± 4.96	8.47 ± 6.43	8.27 ± 7.22	2.61 ± 1.79	4.02 ± 2.27
ROI-wise	29.57 ± 17.61	30.91 ± 23.52	17.35 ± 10.77	24.79 ± 17.88	19.86 ± 18.89	32.22 ± 28.89	4.52 ± 3.97	4.94 ± 2.52
p-value	vw < rwp = 0.001		vw < rwp = 5.3e-5		vw < rwp = 0.005		vw < rwp = 0.1	

Grey matter								
	F		$D^{*}$		$F\cdot D^{*}$		D	
CV [%]	One-step	Two-step	One-step	Two-step	One-step	Two-step	One-step	Two-step
Voxel-wise	11.03 ± 9.01	13.95 ± 13.92	9.05 ± 5.45	16.43 ± 7.80	8.80 ± 10.01	10.07 ± 9.39	2.20 ± 1.44	3.29 ± 2.13
ROI-wise	24.76 ± 15.26	18.36 ± 17.76	30.60 ± 31.39	53.36 ± 28.38	17.85 ± 8,55	29.65 ± 19.10	6.31 ± 3.15	3.39 ± 2.42
p-value	vw < rwp = 0.07		vw < rwp = 0.0003		vw < rwp = 0.002		vw < rwp = 0.01	

(B) ICC values obtained with the voxel-wise and ROI-wise fits using the one-step and two-step algorithms in the white and grey matter.								
White matter								
	F		$D^{*}$		$F\cdot D^{*}$		D	
ICC [-]	One-step	Two-step	One-step	Two-step	One-step	Two-step	One-step	Two-step
Voxel-wise	0.74	0.71	0.94	0.82	0.94	0.95	0.97	0.90
ROI-wise	0.49	0.53	0.82	0.77	0.88	0.89	0.90	0.86

Grey matter								
	F		$D^{*}$		$F\cdot D^{*}$		D	
ICC [-]	One-step	Two-step	One-step	Two-step	One-step	Two-step	One-step	Two-step
Voxel-wise	0.42	0.48	0.78	0.51	0.87	0.88	0.97	0.90
ROI-wise	0.69	0.53	0.89	0.09	0.76	0.08	0.59	0.88

Values of (A) within-subject coefficients of variation (CV) averaged across subjects, and (B) ICC for the different IVIM parameters with the voxel-wise and ROI-wise fits using the one-step and two-step algorithms, in the white matter (WM) and grey matter (GM).

#### 3.3.2 Comparing one-step with two-step fitting algorithms

**Fig. 3.**
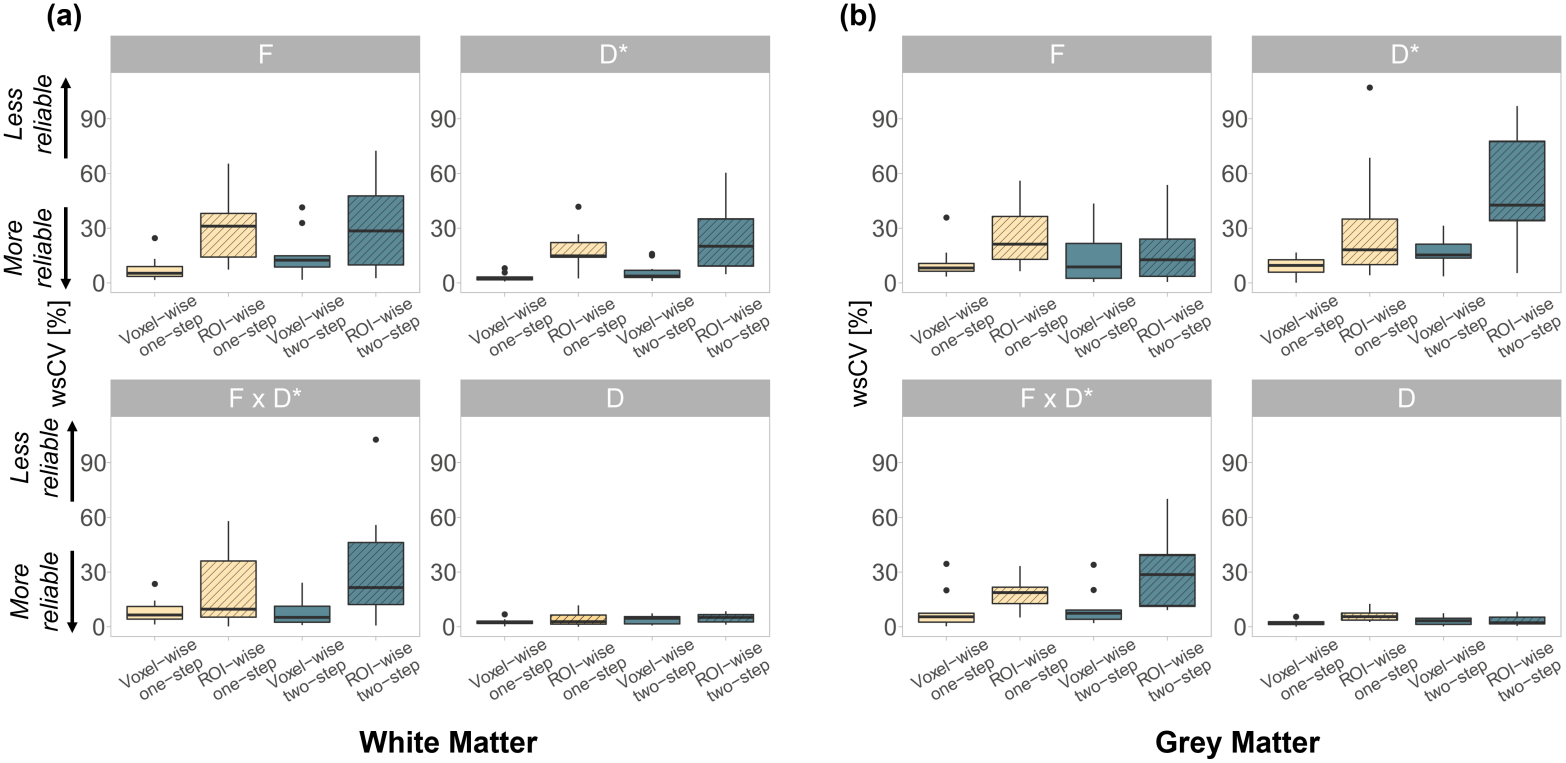
Within-subject coefficients of variation (wsCV) of the IVIM parameters obtained with the voxel-wise and ROI-wise fits, using the one-step and two-step algorithms in the (a) white matter and (b) grey matter. The box extends from the 1^st^to the 3^rd^quartiles, whiskers extend to the smallest and largest value no further than 1.5 times the inter-quartile range, while dots represent outlying values beyond the end of the whiskers.

**Fig. 4.**
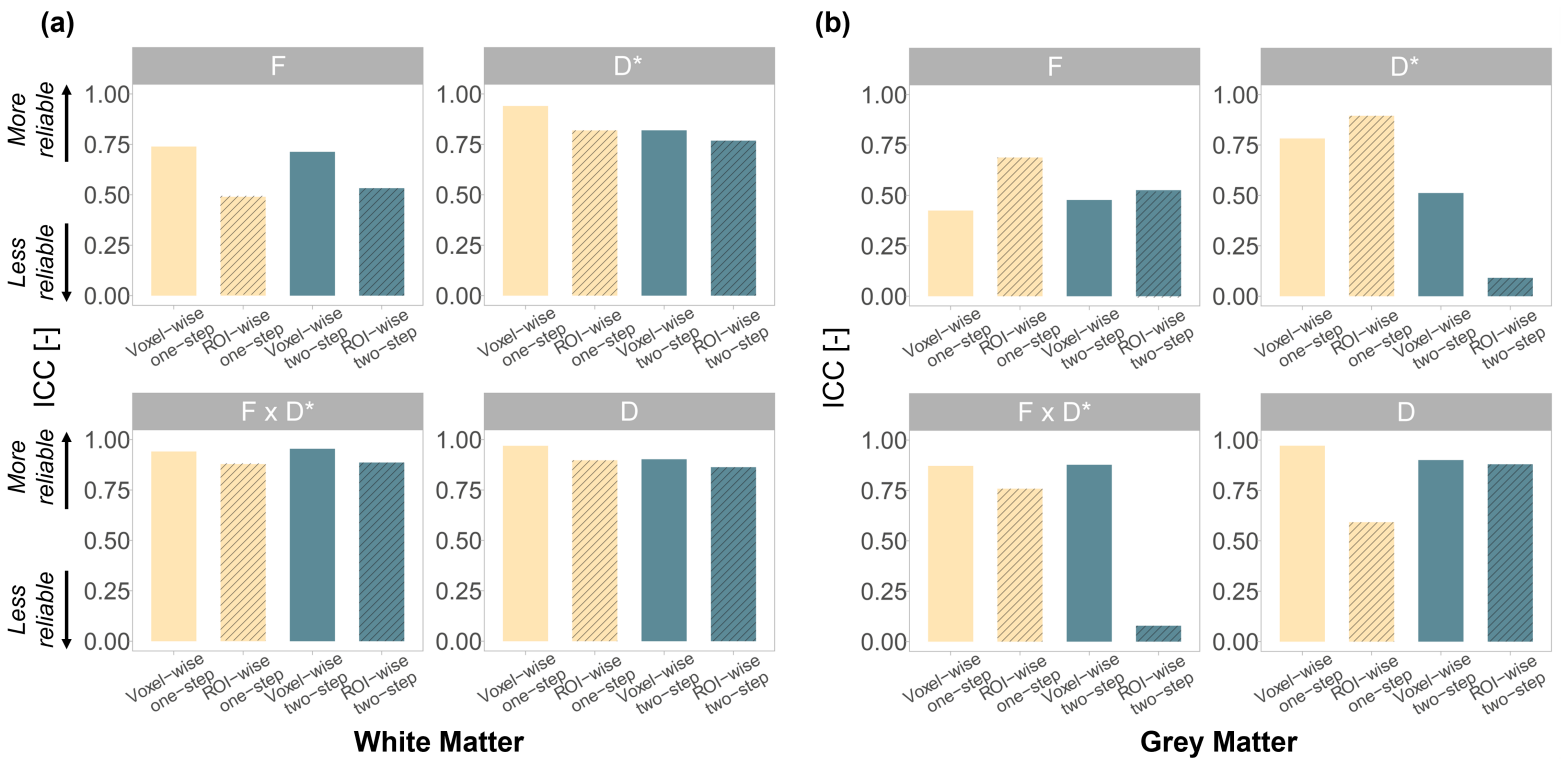
ICC values of the IVIM parameters obtained using the voxel-wise and ROI-wise fits and both the one-step and two-step algorithms in the (a) white matter and (b) grey matter.

While the one-step fitting approach resulted in lower CVs compared to the two-step approach, this difference was not statistically significant. The diffusion coefficient (D) showed the lowest CVs in all fitting scenarios (Fig. 3). The ICC values (Fig. 4) obtained in the white matter were higher with the voxel-wise one-step fitting approach (ranged from 0.74 to 0.97) compared to all other tested scenarios, except for the$F\cdot D^{*}$parameter (ICC: 0.95 vs. 0.94 with the voxel-wise one-step approach). In the grey matter, higher ICC values were obtained forFand$D^{*}$using the ROI-wise fit, whereas voxel-wise yielded better ICC for$F\cdot D^{*}$andD.

**Fig. 5.**
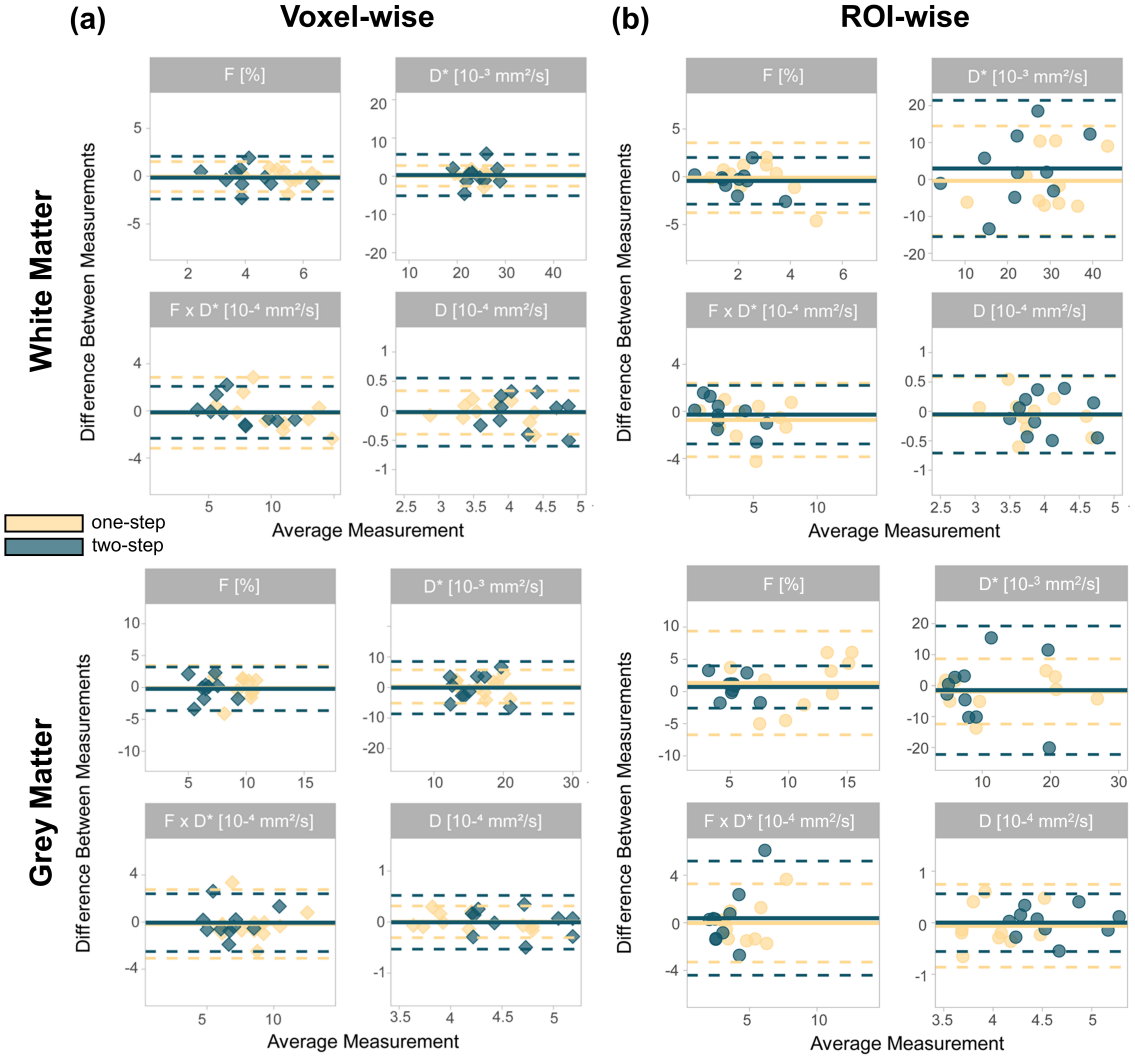
Bland-Altman plots of the IVIM parameters in the white matter (first row) and grey matter (second row) obtained using the (a) the voxel-wise and (b) ROI-wise fit with the one-step and two-step algorithms. The bold lines represent the mean difference between the two sessions (bias), and dashed lines represent the 95% limits of agreement of the mean difference.

The Bland-Altman analysis yielded bias and limits of agreement, which are reported for both voxel-wise and ROI-wise fits using the one-step and two-step algorithms inSupplementary Table 2. The Bland-Altman plots revealed narrower limits of agreement using the one-step method compared to the two-step when fitting the signal in a voxel-wise manner, except for$F\cdot D^{*}$, in the white and grey matter (Fig. 5).

**Fig. 6.**
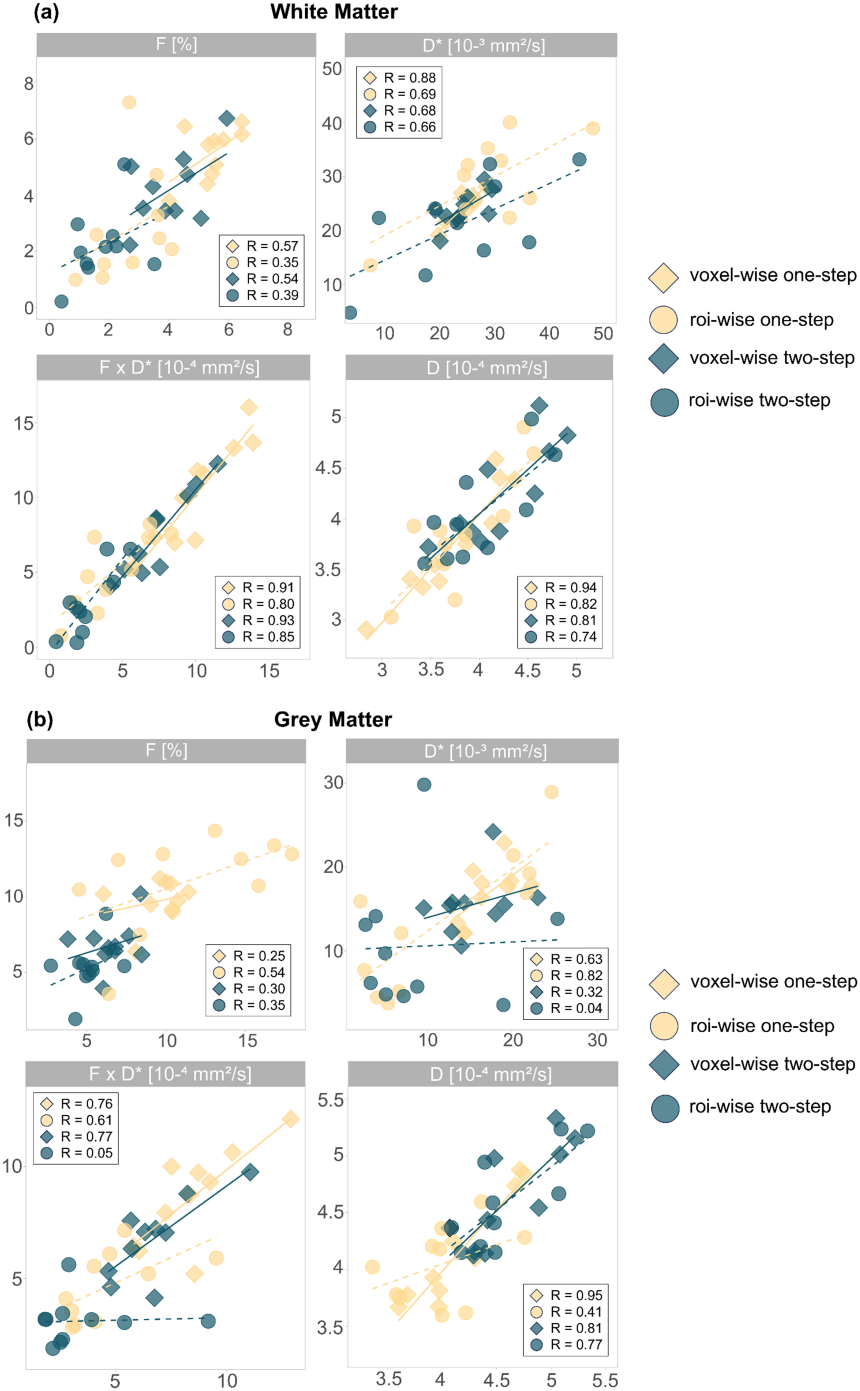
Scatterplots of the IVIM parameters and linear correlations between the first and second sessions in the (a) white matter and (b) grey matter with the voxel-wise fit (bold line) and the ROI-wise fit (dashed line), using the one-step and two-step algorithms, along with the Pearson correlation coefficient R.

IVIM parameters showed good correlations between the first and second scan using the voxel-wise one-step fitting approach for$D^{*}$,$F\cdot D^{*}$, andDin the white matter (Pearson’s correlation coefficient ≥ 0.88) (Fig. 6). The correlations were overall stronger using the voxel-wise fit compared to ROI-wise, and with the one-step approach compared to the two-step one. In general,Fshowed a more moderate inter-session correlation with all fitting approaches (WM: 0.35 ≤ R ≤ 0.57, GM: 0.25 ≤ R ≤ 0.54).

## 4 Discussion

This study showed high repeatability and reliability of IVIM parameters calculated in the cervical cord applying four different fitting approaches. The voxel-wise fitting approach demonstrated higher within-subject repeatability (i.e., lower CVs and higher ICC values) for IVIM parameters compared to the ROI-wise method. While the one-step tended to improve the repeatability of IVIM parameters compared with the two-step method, the difference was not found significantly different.

### 4.1 B-value sampling scheme

The b-value sampling scheme was defined using 14 b-values ranging from 0 to 650 s/mm^2^with an increment of 50 s/mm^2^(Lévy et al., 2021). The highest b-value was set to 650 s/mm^2^as the coefficient of variation across repetitions forDexceeded 50% above that threshold, based on results from a previous feasibility study (Lévy et al., 2021). The constant linear increment of 50 s/mm^2^was chosen to sample the perfusion compartment of the decay curve as evenly as the diffusion compartment. Finally, the cut-off b-value was set to 400 s/mm^2^to ensure complete elimination of perfusion effects in the remaining signal at high b-values.

### 4.2 IVIM parameters in the cervical cord and repeatability

#### 4.2.1 IVIM values and comparison to literature

Microvascular volume fraction (F) maps visually showed higher intensity in the anterior and intermediate grey matter of the cord, which is in line with microangiography of the cord vasculature (Thron, 2016). Values of IVIM parameters varied to a great extent across fitting algorithms, as observed previously in other organs (Gurney-Champion et al., 2018;Spinner et al., 2021), underlining the importance to characterize the repeatability of each acquisition protocol, fitting procedure, in a specific organ of interest, before translation to clinics.

The values ofFobtained from the different fitting configurations fall within the range of what has been previously reported in the brain grey and white matter (Bertleff et al., 2017;Bisdas & Klose, 2015;Federau et al., 2015;Rydhög et al., 2014), which are expected to have similar level of perfusion as the spinal cord (Duhamel et al., 2008). While$D^{*}$values were slightly higher than those reported in the cervical cord and brain in general, the grey matter values were similar to those reported in one brain study (Federau et al., 2015).

$F\cdot D^{*}$values were similar to those obtained in the cervical cord at 7T (Lévy et al., 2020), as were the values ofDin the grey and white matter. TheDvalues were on the lower end of the typical radial diffusivity range reported in DTI studies of the cervical cord (Al-shaari et al., 2024;Cohen-Adad et al., 2011;Grabher et al., 2016). This discrepancy is likely becauseD, derived from IVIM modeling, does not account for perfusion effects, whereas the apparent diffusion coefficient (ADC) incorporates perfusion contributions. White matter showed lowerDvalues than the grey matter, in line with the longitudinal orientation of the fibers in the white matter.

#### 4.2.2 Comparing voxel-wise and ROI-wise fitting approaches

The ROI-wise method resulted in lowerFcompared to the voxel-wise method (range of average values with ROI-wise: 1.73–2.76%; with voxel-wise: 4.03–5.49%) and more variation in$D^{*}$(range of average values with ROI-wise: 21.23–29.57 × 10^-3^mm^2^/s; with voxel-wise: 24.07–24.41 × 10^-3^mm^2^/s), including one-step and two-step, in the white matter. This finding suggests a higher variability in the signal attenuation at lower b-values and demonstrates the importance of the fit choice to model the perfusion contribution to the signal. The tissue diffusion coefficient (D) remained similar in both the voxel-wise and ROI-wise fitting approaches, attesting of the robustness of the estimation ofDto the choice of the fitting method (Andreou et al., 2013).

While the ROI-wise approach led to better R^2^values of the fitting procedure, it showed lower repeatability performance compared to the voxel-wise fit, with significantly higher within-subject CVs (WM:F: p = 0.001,$D^{*}$: p = 5.3e-5,$F\cdot D^{*}$: p = 0.005; GM:$D^{*}$: 0.0003,$F\cdot D^{*}$: p = 0.002,D: p = 0.01) and lower ICC values, indicating the sensitivity of the fit quality on the fitting approach. Of note, the ROI-wise two-step led to low ICC values in the grey matter, indicating the low repeatability of this approach to evaluate$D^{*}$and$F\cdot D^{*},$which could also be observed by the low Pearson R correlation coefficients (Fig. 6). The higher variability of the ROI-wise fitting approach may be due to varying IVIM values within the region where the signal is averaged across. Spinal cord vascularization is known to show substantial variation within subregions of the cord, with noticeable differences between areas such as the ventrolateral funiculi and dorsal columns (Thron, 2016). The ROI-wise approach may be less robust to those local heterogeneities, thereby contributing to the lower reliability observed compared to the voxel-wise fitting method. Additionally, partial volume effects between grey matter, white matter, and CSF regions, as well as between different vessel sizes (capillaries to arteries) may lead to more variability. The theoretical advantage of the ROI-wise fitting approach is the mitigation of the noise at the input of the fitting algorithm. However, given the large number of repetitions used for each b-value (20/b-value), the SNR is unlikely to be a limitation for the voxel-wise fitting approach, as observed with the high range of SNR values obtained, which were of a similar range of SNR values obtained at 7T in a previous study (Lévy et al., 2020).

The white matter showed better repeatability measures compared to the grey matter, using the voxel-wise fit. This is also supported by lower average CVs, higher ICC values, and smaller mean bias with narrower limits of agreements in the Bland-Altman analysis. The results are in line with the strong linear correlation coefficients between the IVIM values in the first and second session. The overall lower repeatability observed in the grey matter is most certainly due to the influence of partial volume effect, small number of voxels, and possible registration imprecisions occurring during the processing steps.

#### 4.2.3 Comparing one-step and two-step fitting algorithms

The differences between the CV values obtained from the one-step and two-step fitting approaches were not found significant. However, previous reports suggested the segmented method (e.g., two-step) to be beneficial at lower SNR forD, whereas the one-step method may yield better reliability in cases of higher SNR (Liu et al., 2023;Park et al., 2017). Those results were also observed in our study, where the one-step fitting approach showed higher reliability (higher ICC) compared to the two-step with the ROI-wise fit, in which the effective SNR is higher due to the average of signal across the region. The difference between the CVs derived from the one-step and two-step methods applying the voxel-wise fitting approach was not significant, yet the one-step led to higher ICC in the white matter compared to the two-step. Individual CV values up to 30% were observed for the voxel-wise one-step fitting method, suggesting that, although the results are promising at the group level, there is room for improvement in terms of confidence at the individual level.

Of note, a clinical study has investigated perfusion impairment in patients with degenerative cervical myelopathy compared to healthy subjects, using the current IVIM protocol (Lebret et al., 2023). The results showed a significant decrease in$D^{*}$and$F\cdot D^{*}$, with an average difference of -9.5% and -15.9% in the white matter and -11.0 % and -14.4% in the grey matter, respectively, at the group level (29 DCM patients, 30 healthy subjects), using the voxel-wise one-step fit. In comparison, the average test-retest differences observed for the parameters in the current study (considering the whole group) were in the range between 4.39% and 11.66% in the white matter, and 12.77% and 12.12% in the grey matter, while the maximum test-retest differences observed at the individual level were 12.15%, 17.37% and 26.67%, and 32.88%, respectively. Moreover, the differences observed in patients give confidence that the estimation method does not compress or restrain the true variations of the parameters while ensuring a good test-retest repeatability.

Furthermore, our results in the cervical cord show high repeatability compared to previous studies on IVIM reliability which used different acquisition protocols and fitting approaches in different organs (Andreou et al., 2013;Jiang et al., 2018;Kang et al., 2017;Koopman et al., 2021;Zhang et al., 2022). A previous study applying IVIM in head and neck tissues reported CVs ofFand$D^{*}$varying between 15.27% and 22.14%, and 29.24% and 41.80%, respectively (Kang et al., 2017). In lung cancer, the mean CVs ofFranged between 36.54% and 38.34%, and 68.59% and 72.62% for$D^{*}$, whileDhad lower CV (11-12%–11.33%) (Jiang et al., 2018). Across previous studies,Dparameter overall showed a better repeatability, which is in line with our results.

We observed an extensive variation in IVIM parameter values depending on the fitting algorithm used, and assessing the accuracy of these values should be the focus of future research. However, the primary focus of our study was to evaluate the repeatability of the IVIM protocol, rather than the absolute accuracy of the parameters, with the aim of providing reliable imaging markers for clinical studies.

Of note, in this study, information was averaged across C1-C3 cervical levels, to reduce variability and noise typically encountered at the single slice level, as the protocol focuses on clinical applications in spinal cord pathologies. Future research would benefit from analyzing slice-wise information, though this approach would necessitate further investigation.

#### 4.2.4 Comparison to other fitting methods

In recent years, extensive effort has been made to investigate alternative approaches for the fitting of IVIM data, such as Bayesian methods (Spinner et al., 2021) and deep learning-based algorithms (Koopman et al., 2021), which have shown great promises in terms of noise robustness and parameter estimation accuracy. The study of those alternative methods was beyond the scope of the current study and would necessitate further technical development.

### 4.3 Limitations

This study has a few limitations. First, the sample size and the number of sample points were rather small, and larger cohorts and more retests are required to provide more accuracy. However, the current cohort size is comparable to previous IVIM reliability studies (Kang et al., 2017;Koopman et al., 2021;Simchick et al., 2022;Zhang et al., 2022). Second, young healthy volunteers (24 years < age < 42 years) were recruited in this study. Repeatability with patients and elderly cohorts can be lower, which should be either accounted for in clinical studies or quantified in subsequent research. Furthermore, the current EPI distortion correction algorithm (topup) was originally optimized for brain imaging. A recent study has highlighted some limitations when applying it to the spinal cord (Schilling et al., 2024), and the potential impact of distortion correction algorithms on repeatability should be kept in mind. However, the use of axial EPI ZOOMit in our acquisition reduces the likelihood of distortions, due to the smaller field of view and encoding matrix. Finally, the nominal acquisition time of the protocol is currently long (ca. 39 minutes) to be transferred to clinical routine. The acquired test-retest IVIM maps are a great opportunity to perform time optimization of the acquisition protocol without compromising the obtained SNR nor the reliability of the IVIM values. Of note, a preliminary analysis explored the impact of the number of averages per b-value (3, 5, and 10) on repeatability (seeSupplementary Fig. 1). Although further in-depth investigation is warranted, the preliminary findings suggest that the use of 10 averages provides a more reliable and robust estimation of the IVIM parameters. It must also be noted that the test-retest variability reported here includes the test-retest variability of the entire post-processing chain, including the automatic segmentation and calculation of the probabilistic masks. The different steps involved are not free from inter-session variations and might contribute to the final test-retest variability of the IVIM values reported in this study.

## 5 Conclusion

Our study showed high repeatability of IVIM parameters in the human cervical cord assessed with four different fitting approaches, comparing voxel-wise vs. ROI-wise fits, and one-step vs. two-step fitting algorithms. The voxel-wise fitting approach showed a higher repeatability of IVIM-derived parameters in the cervical cord, compared to the ROI-wise fit approach, independently of using the one-step or two-step algorithms. The present work provides hereby reference values for future clinical investigations of the perfusion integrity in the cervical cord in different neurodegenerative pathologies.

## Supplementary Material

Supplementary Material

## Data Availability

Anonymized datasets used and analyzed during the current study are available from the corresponding author on reasonable request after completing a Data Sharing Agreement. The pipeline code is available in a public repository (https://github.com/slevyrosetti/ivim-toolbox).
