# Supplementary material for: Test-retest repeatability of intravoxel incoherent motion (IVIM) measurements in the cervical cord
Source: Imaging Neurosci (Camb). 2025 Feb 10;3:imag_a_00468. doi: 10.1162/imag_a_00468 (PMC12319757; doi:10.1162/imag_a_00468)
Supplement: Supplementary Material [file imag_a_00468-supp.pdf]

Supplementary Table 1: Mean and standard deviation (SD) values of R^2 of the fit across participants obtained in the (A) white matter and (B) grey matter using the (i) voxel-wise and (ii) ROI-wise fits, using the one-step and two-step algorithms in each scanning session. Results are reported for each of the three diffusion-encoding directions (180°, 60°, and -60°).

(A) White Matter

(i) voxel-wise

Session 1			
Mean ± SD	180°	60°	-60°
One-step	96.28 ± 3.29	93.88 ± 5.06	95.35 ± 2.77
Two-step	91.74 ± 8.22	89.12 ± 7.76	89.74 ± 7.55

Session 2			
	180°	60°	-60°
One-step	96.03 ± 1.98	95.65 ± 2.27	95.06 ± 3.65
Two-step	91.80 ± 6.43	88.80 ± 8.81	88.67 ± 10.54

(ii) ROI-wise

Session 1			
Mean ± SD	180°	60°	-60°
One-step	99.67 ± 0.33	99.16 ± 0.97	99.38 ± 0.53
Two-step	99.45 ± 0.80	98.60 ± 1.31	98.88 ± 1.23

Session 2			
	180°	60°	-60°
One-step	99.51 ± 0.31	99.19 ± 0.51	99.10 ± 0.56
Two-step	99.24 ± 0.64	98.05 ± 2.22	98.06 ± 2.29

(B) Grey Matter

(i) voxel-wise

Session 1			
Mean ± SD	180°	60°	-60°
One-step	98.84 ± 0.59	97.77 ± 2.47	98.48 ± 0.88
Two-step	97.95 ± 1.36	97.45 ± 1.33	97.31 ± 1.95

Session 2			
	180°	60°	-60°
One-step	98.50 ± 0.50	98.32 ± 0.78	98.28 ± 0.98
Two-step	97.62 ± 1.10	96.58 ± 3.05	96.76 ± 2.84

(ii) ROI-wise

Session 1			
Mean ± SD	180°	60°	-60°
One-step	99.79 ± 0.15	99.61 ± 0.54	99.67 ± 0.27
Two-step	99.77 ± 0.16	99.56 ± 0.54	99.65 ± 0.31

Session 2			
	180°	60°	-60°
One-step	99.72 ± 0.19	99.61 ± 0.26	99.65 ± 0.21
Two-step	99.68 ± 0.22	99.52 ± 0.28	99.57 ±0.24

Supplementary Table 2: Limits of agreement and bias of the Bland-Altman analysis. Lower bounds and upper bound of the 95% limits of agreement, and bias for the IVIM parameters using the (A) voxel-wise and (B) ROI-wise fits.

(A) Bland-Altman analysis values obtained with the voxel-wise fit, using the one-step and two-step algorithms in the white and grey matter.

White Matter

	$\boldsymbol{F}$ [%]			$\boldsymbol{D}^*$ [mm²/s ×10⁻³]			$\boldsymbol{F \cdot D}^*$ [mm²/s ×10⁻⁴]			$\boldsymbol{D}$ [mm²/s ×10⁻⁴]		
	Lower bound	Upper bound	Bias	Lower bound	Upper Bound	Bias	Lower bound	Upper bound	Bias	Lower bound	Upper bound	Bias
One-step	-1.60	1.52	-0.041	-2.58	2.77	0.094	-3.18	2.85	-0.17	-0.40	0.34	-0.028
Two-step	-2.38	2.08	-0.15	-5.11	5.73	0.31	-2.33	2.08	-0.13	-0.60	0.56	-0.022

Grey Matter

	$\boldsymbol{F}$ [%]			$\boldsymbol{D}^*$ [mm²/s ×10⁻³]			$\boldsymbol{F \cdot D}^*$ [mm²/s ×10⁻⁴]			$\boldsymbol{D}$ [mm²/s ×10⁻⁴]		
	Lower bound	Upper bound	Bias	Lower bound	Upper Bound	Bias	Lower bound	Upper bound	Bias	Lower bound	Upper bound	Bias
One-step	-3.65	3.43	-0.11	-5.12	5.75	0.31	-3.07	2.76	-1.56	-0.31	0.32	0.0048
Two-step	-3.62	3.21	-0.21	-8.62	8.48	-0.70	-2.50	2.41	-0.48	-0.53	0.52	-0.0041

(B) Bland-Altman values obtained with the ROI-wise fit, using the one-step and two-step algorithms in the white and grey matter.

White Matter												
	$\boldsymbol{F}$ [%]			$\boldsymbol{D}^*$ [mm²/s ×10⁻³]			$\boldsymbol{F} \cdot \boldsymbol{D}^*$ [mm²/s ×10⁻⁴]			$\boldsymbol{D}$ [mm²/s ×10⁻⁴]		
	Lower bound	Upper bound	Bias	Lower bound	Upper Bound	Bias	Lower bound	Upper bound	Bias	Lower bound	Upper bound	Bias
One-step	-3.75	3.56	-0.095	-15.21	14.49	-3.59	-3.85	2.40	-0.73	-0.71	0.59	-0.062
Two-step	-2.87	2.01	-0.43	-15.50	21.44	2.97	-2.76	2.21	-0.28	-0.71	0.61	-0.051

Grey Matter												
	$\boldsymbol{F}$ [%]			$\boldsymbol{D}^*$ [mm²/s ×10⁻³]			$\boldsymbol{F} \cdot \boldsymbol{D}^*$ [mm²/s ×10⁻⁴]			$\boldsymbol{D}$ [mm²/s ×10⁻⁴]		
	Lower bound	Upper bound	Bias	Lower bound	Upper Bound	Bias	Lower bound	Upper bound	Bias	Lower bound	Upper bound	Bias
One-step	-7.64	8.47	0.41	-12.43	8.66	-1.88	-3.31	3.27	-0.018	-0.86	0.74	-0.059
Two-step	-3.49	3.07	-0.21	-22.25	19.29	-1.48	-4.42	5.18	0.38	-0.56	0.56	0.022

Supplementary Figure 1

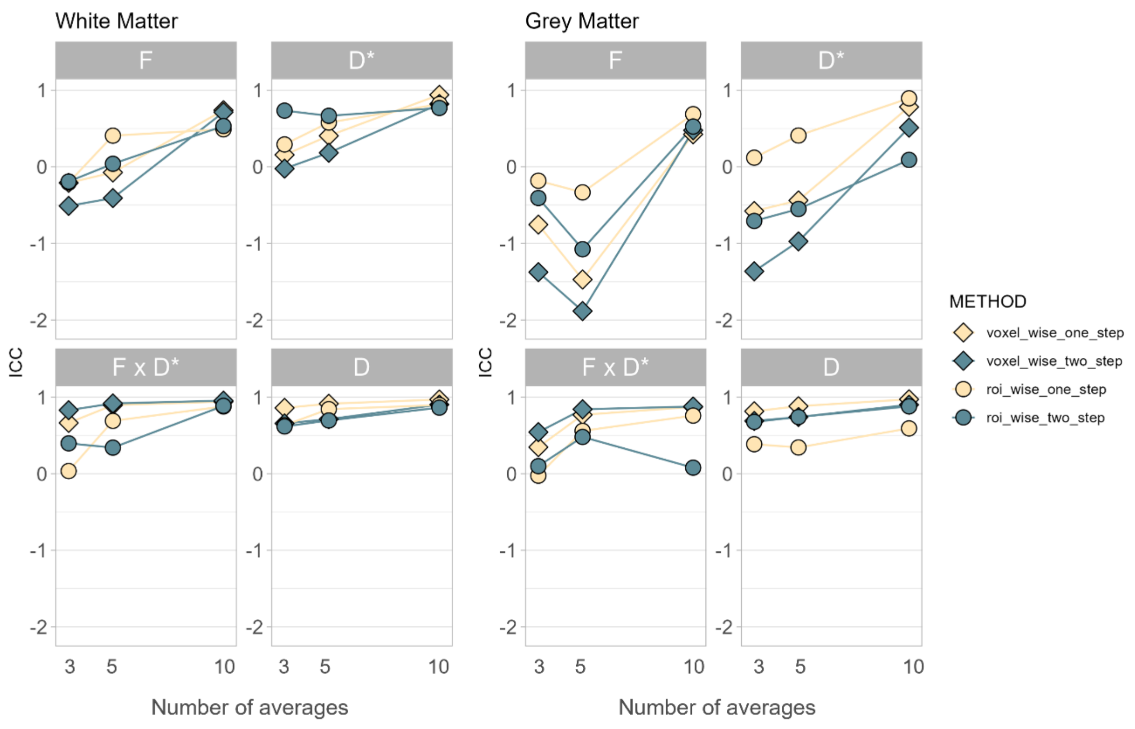

Supplementary Figure 1: ICC dependence on the number of averages per b-value. ICC values obtained with three different numbers of averages per b-value (i.e., 3, 5, and 10) are displayed for the four IVIM parameters (F, D^*, $F \cdot D^*$, and D) and the four investigated fitting algorithms (voxel-wise vs. ROI-wise, and one-step vs. two-step) in the white matter (left panel) and grey matter (right panel).
